# Clinical comparative study of single-use and reusable digital flexible ureteroscopy for the treatment of lower pole stones: a retrospective case-controlled study

**DOI:** 10.1186/s12894-024-01541-5

**Published:** 2024-07-18

**Authors:** Qiang Jing, Fan Liu, Xiaobin Yuan, Xuhui Zhang, Xiaoming Cao

**Affiliations:** Department of Urology, First Hospital of Shanxi Medical University, No. 85, Jiefang South Road, Taiyuan, Shanxi 030001 China

**Keywords:** Lower pole stone, Single-use flexible ureteroscopy, Reusable flexible ureteroscopy, Efficacy

## Abstract

**Objectives:**

To compare the clinical efficacy and safety of single-use and reusable digital flexible ureteroscopy for the treatment of lower pole stones.

**Methods:**

We enrolled 135 patients underwent reusable flexible ureteroscopy (FURS) and 78 patients underwent single-use digital FURS. Demographic, clinical variables, anatomical parameters of the lower calyx and perioperative indicators were compared in the two groups.

**Results:**

Thirty-six patients in the infundibuloureter angle (IPA) < 45° subgroup had a mini-percutaneous nephrolithotomy (mini-PCNL), including 25 patients in the reusable FURS group and 11 patients in the single-use FURS group. The demographic and clinical variables in the two FURS groups were comparable. There was no statistical difference in the success rate of stone searching (*P* > 0.05). In terms of the success rate of lithotripsy, there was also no statistical difference in the IPA ≥ 45° subgroup (*P* > 0.05), whereas single-use FURS was superior in the IPA < 45° subgroup (χ2 = 6.513, *P* = 0.011). The length of the working fiber in the reusable FURS and single-use FURS groups was 3.20 ± 0.68 mm and 1.75 ± 0.47 mm, respectively (t = 18.297, *P* < 0.05). The use of a stone basket in the reusable FURS (31/135, 23.0%) was significantly higher than that in the single-use FURS (8/78, 10.3%) (χ2 = 5.336, *P* = 0.021). Compared with the reusable FURS group, the single-use FURS group had shorter operation times (*P* < 0.05) and higher stone-free rate (SFR) (χ2 = 4.230, *P* = 0.040). There was no statistical difference in the intraoperative transfer of mini-PCNL and postoperative complications between the two groups (*P* > 0.05).

**Conclusions:**

Single-use and reusable FURS are alternative methods for removal of lower pole stones (i.e., 2 cm or less). Single-use FURS has a high success rate of lithotripsy, shorter operation time, and high stone-free rate.

## Introduction

With the continuous improvement in endoscopic technology and the development of corollary equipment, such as ureteric access sheaths and stone baskets, flexible ureteroscopy (FURS) combined with holmium laser lithotripsy has become an important treatment for upper urinary calculi with a diameter of ≤ 2.0 cm [1]. The first flexible endoscope was used in 1965 by Marshall, designed by Curtis and Hirshowitz [2, 3]. The FURS had advantages that required further development, as it allowed access to the renal cavities. Fibre-optic FURS were developed primarily as a response to concerns that the rigid URS (rURS) could cause damage to the urothelium when accessing the upper ureter [4]. Introduced in 2006, the first digital ureteroscope was the Invisio ® DUR ® -D (Olympus) [5]. However, the effectiveness of lithotripsy for lower pole stones is not ideal due to the influence of the anatomical structure of the lower calyx. Studies have shown that the infundibuloureter angle (IPA) directly affects the curative effect of FURS combined with holmium laser lithotripsy [6]. A systematic review of the literature showed that with an acute IPA (< 30°), the duration of the operation and a larger stone size were significant predictors of FURS failure. However, the placement of the ureteric access sheath, or infundibular width (IW) and length (IL) did not influence treatment outcomes [7]. Due to updating of equipment and an improvement in clinical techniques, lower pole stones with an IPA < 30° are not contraindicated for reusable FURS [8]. However, the damage rate of the lens during the FURS operation is high, and it has been reported that flexible ureteroscopes need to be repaired after 6–21 operations [9], limiting their clinical application and resulting in high maintenance costs. In recent years, single-use FURS have gradually been used in clinical practice and have achieved the same efficacy as reusable FURS [10, 11]. The deflection performance of single-use FURS has been shown to be superior to reusable digital or fiber FURS and even if a 275 μm fiber is inserted, the deflection can reach 297° [12]. In addition, single-use FURS does not risk damage to the equipment, with these advantages overcoming the corrupt practice of reusable FURS being used repeatedly for the treatment of the lower pole stones. This retrospective study analyzed the clinical data of patients with a lower pole stone, and compared the efficacy and safety of single-use and reusable FURS in the treatment of these stones.

## Patients and methods

### Patients

Patients with a lower pole stone admitted to the First Hospital of Shanxi Medical University between April 2018 and October 2021 were enrolled in the study. This included 135 patients who underwent reusable FURS combined with holmium laser lithotripsy (R-FURS group) and 78 patients who underwent single-use FURS combined with holmium laser lithotripsy (SU-FURS group). The following parameters were recorded in all the patients: age, body mass index (BMI), gender, stone side, location, diameter, and density, IPA, IW, and IL. Related lines were drawn on the IVP image, and the IPA were automatically measured using angle meter software(Syngo.plaza-VB20A_HF05). IW and IL were automatically measured using range measuring software(Syngo.plaza-VB20A_HF05).All the procedures performed in the study involving patients were in accordance with the revised 2013 version of the Declaration of Helsinki. The study was approved by the institutional ethics board of First Hospital of Shanxi Medical University(No.K-K133).

#### Inclusion criteria

The information of patients were collected through hospital record; Unenhanced CT examination that confirmed the presence of a unilateral, single lower pole stone with a maximum diameter ≤ 2.0 cm; no history of ureteral stricture or surgery; normal function of the heart, lung, liver, and kidney; no urinary tract infection or an infection under preoperative control.

#### Exclusion criteria

Previous ipsilateral treatment with extracorporeal shock wave lithotripsy or endoscopy; patients with bilateral renal stones undergoing flexible ureteroscopic lithotripsy at the same time; patients with multiple kidney stones or ureteral stones; patients with a ureteral stricture or a history of surgery; isolated kidney; patients with severe underlying diseases, abnormal coagulation profile, cardiopulmonary insufficiency, or American Society of Anesthesiology(ASA)Grade III or above.

All patients underwent preoperative blood and urine analysis, urine bacterial culture, coagulation function, liver and kidney function, and other routine laboratory tests. An unenhanced urinary CT scan and intravenous pyelography (IVP) examination were performed, with the density and diameter of the calculi and IPA, IL, and IW obtained from the imaging results. Patients with a negative urine bacteria culture were treated with preoperative prophylactic antibiotics according to expert advice on the application of perioperative antibiotics in patients with upper urinary calculi. Patients with a positive urine bacterial culture were treated with antibiotics for more than one week according to the results of urine culture bacteriology and drug sensitivity test obtained before surgery. Reexamination confirmed that the urine bacterial culture was negative and the urine white blood cell count had improved significantly.

### Surgery

All patients received general anesthesia and were then placed in the lithotomy position. A F_8_ rigid ureteroscope was inserted through the urethra to enter the bladder and ensure no stones or tumors were present. The ureter opening of the bladder on the affected side was located and then a guide wire (Bard Medical Technology Co. Ltda., Shanghai, China) was inserted through the ureteroscope. Under the guidance of the guide wire, no obvious stenosis was found in the middle and lower segment of ureter, with the indwelling guide wire used to exit the rigid ureteroscope. Using the indwelling guide wire, a ureteral access sheath (10/12 Fr Flexor® Cook® Medical, USA) was placed into the ureter, and then either a single-use FURS (Uscope 3022A®, Zhuhai Pusen Medical Technology Co. Ltd., Zhuhai, China) or a reusable FURS (URF-V®, Olympus, Japan) was inserted into the ureteral access sheath. After entering the renal pelvis and calyces and identifying a calculi in the lower calyces, a 272 μm holmium laser fiber (Shanghai Raycone Laser Technology Co. Ltd,1.0J,30Hz) was used to pulverize the calculi. In order to improve the efficiency of lithotripsy and stone removal, stone baskets were used in both methods. An indwelling F_6_ ureteral stent (Bard Medical Technology Co. Ltd., Shanghai, China) was inserted routinely and an indwelling urethral catheter placed in the bladder. All patients were operated on by a senior surgeon and followed up by junior doctors.

### Assessment parameters

The operative time was defined as the period from insertion of the digital flexible ureteroscope into the ureteral access sheath to the departure of the lens after lithotripsy. The success rate of stone searching was defined as the digital flexible ureteroscope successfully entering the lower renal calyx to locate the lower pole stone, while the success rate of lithotripsy was defined as the flexible ureteroscope combined with the holmium laser fiber successfully penetrating the lower calyceal and crushing the calculi. The working fiber length refers to the distance from the holmium laser fiber tip placed in the operation hole of the ureteroscope to the endoscope lens during the operation. Surgical complications, including fever, lumbago, and hematuria were recorded. The duration of postoperative hospital stay was collected from the inpatient records of the patients. A further CT was performed one month after discharge to calculate the stone-free rate, defined as no residual fragments or residual fragments < 3 mm and no clinical symptoms. All patients were re-examined by the same surgeon.

### Statistical analysis

The data were analyzed using SPSS 22.0 for Windows (IBM, Armonk, NY, USA). Potential confounding variables included age, sex, BMI, stone side, stone diameter, stone density, preoperative stenting, IPA, IL, IW. Selected characteristics between SU-FURS and R-FURS were compared using t-tests when conforming to normal distribution and the Wilcoxon rank sum test when not conforming to normal distribution. Categorical data were expressed as case number and frequencies and were analyzed using the chi-square test or Fisher exact test. Statistically significant differences were defined as two-sided *P* values < 0.05.

## Results

Of the 213 patients with a lower pole stone, retrograde intrarenal surgery (RIRS) failed in 36 cases even when combined with a stone basket due to the difficult anatomical structure of the lower calyx or stone entrapment. This resulted in 25 cases in the reusable FURS group and 11 cases in the single-use FURS group requiring a mini-percutaneous nephrolithotomy (mini-PCNL). Importantly, the IPA was < 45° in all 36 patients who transferred to a mini-PCNL. Demographic and clinical variables as well as in important parameters such as IPA, IW, and IL for evaluating the anatomical structure of the lower calyx were comparable in the two groups (Table 1).

**Table 1:** Demographic and baseline features prior to surgery

Variables	SU-FURS (*n* = 78)	R-FURS (*n* = 135)	t/χ^2^	*P* value
Age	41.31 ± 13.86	38.91 ± 10.41	-1.326	0.187
Sex (Male/Female,n)	56/22	97/38	0.000	0.993
BMI(kg/m^2^)	22.27 ± 4.26	23.53 ± 5.51	0.382	0.703
Stone side(Left/Right,n)	43/35	76/59	0.027	0.869
Stone diameter(mm)	1.36 ± 0.40	1.43 ± 0.33	1.385	0.168
Stone density(HU)	885.87 ± 237.39	821.58 ± 290.19	-1.752	0.081
Preoperative stenting(n,%)	9(14.1)	24(17.8)	0.486	0.486
IPA(°)				
< 45°	34.56 ± 5.85	35.37 ± 4.94	0.750	0.455
≥ 45°	52.67 ± 3.94	53.38 ± 6.04	0.751	0.454
IL(mm)	32.48 ± 3.74	33.57 ± 4.45	1.909	0.058
IW(mm)	4.85 ± 0.81	4.81 ± 1.04	-0.296	0.767

*R-FURS* Reusable flexible ureteroscopy, *SU-FURS* Single-use flexible ureteroscopy, *BMI* Body mass index, *IPA* Infundibuloureter angle, *IL* Infundibular length, *IW* Infundibular width

There was no statistical difference in the success rate of stone searching between the reusable and the single-use FURS groups regardless of whether the IPA was less or greater than 45° (*P* > 0.05). In term of the success rate for lithotripsy, there was no significant difference in the IPA ≥ 45 subgroups (*P* > 0.05), although in the IPA < 45°subgroup, single-use FURS was superior to reusable FURS (χ^2^ = 6.513, *P* = 0.011). The length of working fiber in the reusable and single-use FURS groups was 3.20 ± 0.68 mm and 1.75 ± 0.47 mm, respectively, with this difference being statistically significant (t = 18.297, *P* < 0.05). The use of a stone basket in the reusable FURS group (31/135, 23.0%) was significantly higher (χ2 = 5.336, *P* = 0.021) than that in the single-use FURS group (8/78, 10.3%). Compared with the reusable FURS group, the single-use FURS group had significantly shorter operation times (*P* < 0.05). There was no statistical difference in the intraoperative transfer to a mini-PCNL and postoperative complications between the two groups (*P* > 0.05). The stone-free rate was calculated by unenhanced CT one month after surgery, and was higher in the single-use FURS group than in the reusable FURS group (χ^2^ = 4.230, *P* = 0.040) (Table 2).

**Table 2:** Comparison of perioperative indicators between reusable FURS group and single-use FURS group

Variables	SU-FURS (*n* = 78)	R-FURS (*n* = 135)	t/χ^2^	*P* value
Success rate of stone searching(n,%)				
IPA < 45°(*n* = 67/36)	31(86.1)	53(79.1)	0.764	0.382
IPA ≥ 45°(*n* = 68/42)	42(100)	66(97.1)	1.258	0.262
Success rate of lithotripsy(n,%)				
IPA < 45°(*n* = 67/36)	21(66.7)	27(40.3)	6.513	0.011^*^
IPA ≥ 45°(*n* = 68/42)	39(90.5)	54(79.4)	2.322	0.128
Transfer of Mini-PCNL(n,%)	11(14.1)	25(18.5)	0.686	0.407
Use of stone basket(n,%)	8(10.3)	31(23.0)	5.336	0.021^*^
Operation time(min)	51.27 ± 13.80	69.50 ± 16.76	8.576	0.000^*^
Postoperative hospital stay(d)	2.86 ± 1.50	3.14 ± 1.37	4.669^a^	0.160
The length of working fiber(mm)	1.75 ± 0.47	3.20 ± 0.68	18.297	0.000^*^
Surgical complications				
Fever (n,%)	13(16.7)	21(15.6)	0.045	0.831
Lumbago(n,%)	11(14.1)	25(18.5)	0.686	0.407
Hemorrhage (n,%)	23(29.5)	48(35.6)	0.819	0.365
SFR (n,%)	69(88.5)	104(77.0)	4.230	0.040^*^

*R-FURS* Reusable flexible ureteroscopy, *SU-FURS* Single-use flexible ureteroscopy, *IPA* Infundibuloureter angle, *SFR* Stone-free rate

^*^P < 0.05

^a^Mann-Whitney U was used based on this variable was not distributed normally

## Discussion

FURS combined with holmium laser lithotripsy has become an important method for the treatment of upper urinary calculi (stone diameter < 2 cm) due to it being minimally invasive and high efficient [13]. However, patients with unfavorable anatomical factors of the lower calyx have lower success rates in FURS and ESWL [14, 15]. A recent study reported that patients with a high IL or a very acute IPA were more likely to require a second procedure that did not appear to influence the rate of complications and ESWL [16]. Liu et al. compared the outcomes of PCNL, FURS, and ESWL in the treatment for lower pole stones and showed that PCNL and FURS had lower retreatment rates, while PCNL had the longest hospital stay [17].

The surgical complication rate is an important indicator for evaluating the safety of surgery. Some scholars have conducted detailed studies on the complications of PCNL and reported that the total complication rate of postoperative infections, bleeding, blood transfusions, and peripheral organ injury was as high as 15%, a rate significantly higher than that of FURS [18]. In recent years, progress in minimally invasive technology and improvements in surgical instrument research and development have resulted in treatment of kidney stones using mini-PCNL achieving a stone clearance rate comparable to that of standard PCNL, with fewer postoperative complications and shorter hospital stays [19]. Coskun and collagues compared mini-PCNL and RIRS for lower pole stones and demonstrated no meaningful difference in stone-free rates between the two groups, although complications such as the use of fluoroscopy, bleeding, and duration of hospital stay were significantly higher in cases treated with mini-PCNL [20].

Since the European Urological Guidelines in 2015 recommended FURS as the first-line treatment for lower pole stones, this procedure has become the most favored method for removing upper urinary calculi by both doctors and patients due to its less invasive nature and lower risk of intraoperative and postoperative bleeding. In-vitro, it appears that single-use FURSs deflect better than their reusable counterparts, reusable FURSs had better vision characteristics than single-use FURSs. Further in-vivo studies might be necessary to confirm these findings [21]. Professor Jonathan Kan and collagues compared single-use and reusable digital flexible ureteroscopy and found the the URF-V2 group had higher visibility scores than the single-use scopes and higher maneuverability. however there were no differences in operative time, rates of relook flexible ureteroscopes, scope failure or complication rates observed [10].

With the popularization of flexible ureteroscopes, researchers have reported that lower pole stones represent the main challenge for FURS combined with holmium laser lithotripsy. An acute IPA makes it difficult for the lens to reach the stone position, leading to a decrease in the efficiency of lithotripsy [6], or even transferring to a PCNL. In our study, we found that the lens could almost enter the lower calyx of patients in the subgroup with an IPA ≥ 45°, whereas in the subgroup with an IPA < 45°, the success rate of intraoperative stone searching of single-use FURSs was higher reusable FURSs and there is no statistical significance. When the 272 μm holmium laser fiber was inserted, its deflection was affected and it could not enter the lower calyx smoothly. The success rate of intraoperative lithotripsy was only 40.3% in the subgroup with an IPA < 45°, with 18.5% patients being transferred to a mini-PCNL, even after the lower pole stone were displaced using a set of stone baskets. Richards et al. noted that an IPA < 45° was associated significantly with the stone-free rate after FURS [22]. This raises the question of how to maximize the flexible characteristics of flexible ureteroscopes in cases with an adverse anatomical structure of the lower pole stone? Inoue and collagues suggested that an acute IPA mainly affected postoperative stone removal rather than intraoperative stone finding and holmium laser lithotripsy [23].

In recent years, the use of single-use, digital flexible ureteroscopes has been described in published literature that has led to their current use in clinical practice. Leveillee et al. reported a case of a 35-year-old female with a lower pole stone, in who a new disposable digital ureteroscope allowed for extreme lower pole access and the use of a 365 μm holmium laser fiber [24]. Single-use digital flexible ureteroscopes have visibility and maneuverability profiles approaching that of reusable digital flexible ureteroscope. There is also evidence that single-use flexible ureteroscopes achieve similar clinical outcomes to the more expensive reusable versions [10, 25], even a higher SFR than reusable FURS [25].

The terminal deflection ability of reusable flexible ureteroscopes will gradually decrease after repeated use or even following maintenance, especially in patients with a complex anatomical structure of the renal inferior calyceal. Single-use FURS not only overcomes the reduction in deflection ability that occurs after considerable use, but also helps doctors deal with concerns about the high cost of repairing damaged devices, and lets them target the lower calyx in cases with an adverse anatomical structure. Both studies compared single-use and reusable FURS in the treatment of lower pole stones and showed that single-use FURS had better deflection compared with reusable FURS, a characteristic conducive to the treatment of lower pole stones with a heavy load [26, 27]. Our study showed that compared with reusable FURS, a single-use FURS had advantages in surgical time and stone clearance rate, entirely due to their superior deflection ability. In addition, the fact that single-use FURS does not involve the risk of equipment damage, these advantages overcome the disadvantages of reusable FURS in the treatment of lower pole stones. Our data showed that single-use and reusable FURS had a comparable success rate for stone searching. Regarding the success rate of lithotripsy, the efficacy of the two treatments was similar in the subgroup with an IPA ≥ 45°, whereas in the subgroup with an IPA < 45°, single-use FURS had several advantages. This solved the “unattainable” dilemma of FURS combined with holmium laser lithotripsy for patients with a lower pole stone. The experimental results shown that SU-FURS have more advantages in handling the lower pole stones, such as success rate of lithotripsy when IPA < 45° and use of stone basket. In addition, SU-FURS was much lighter than R-FURS in weight, decreased the fatigue of surgeon and shorten the operation time.

**Fig. 1:**
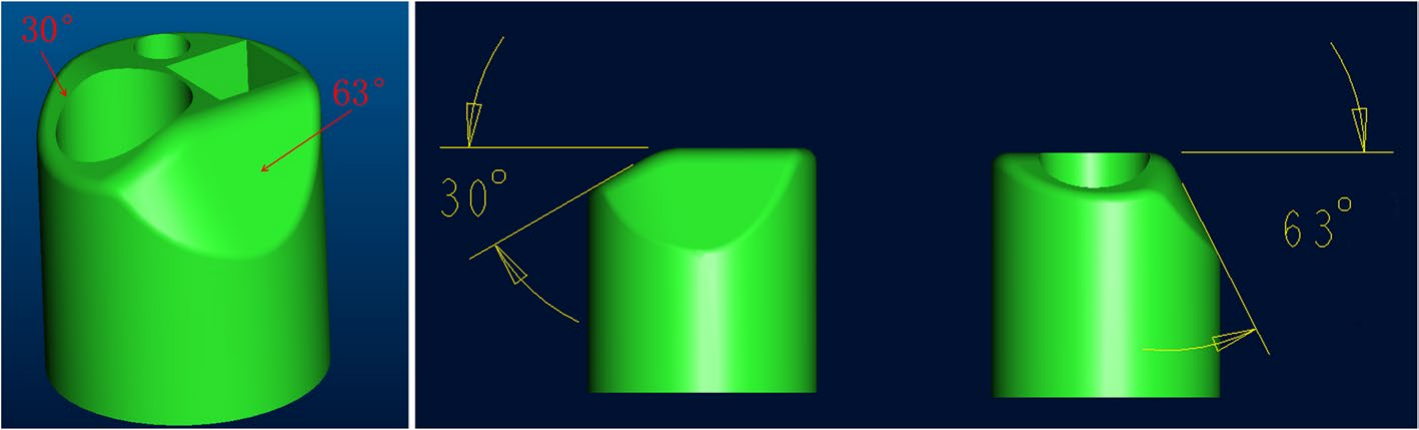
Schematic diagram of tip design of single-use FURS. The single-use flexible ureteroscope has a 30° bevel at the operation hole. On the side of the operation hole, there is a 63^o^ bevel. This characteristic is a consequence of the unique design of the tip of the single-use FURS, and can ensures that the length of the working fiber in single-use FURS can be kept shorter

On the other hand, placing the laser fiber through the operation hole of the reusable FURS requires the tip to reach a quarter of the endoscope screen to avoid damage to the lens caused by cavitation bubbles produced during laser excitation [28]. However, no deterioration in the quality of the image and illumination was observed when firing the laser at every fiber tip to the working channel position (10 mm to − 2 mm) for 10 s. Even when firing for 5 min at a distance of 0 mm (i.e., fiber tip even to the working channel outlet), no impact on image and illumination quality was observed [12]. This characteristic is a consequence of the unique design of the tip of the single-use FURS (Fig. 1), which ensures accurate lithotripsis in cases with difficult anatomical structures of the lower pole stone. We often encountered a challenge when the fiber was located at the edge of the stone edge and could not effectively break-down the stone because of a problem with the length of the working fiber, resulting in the fiber being out of view when using reusable FURS in clinical practice. However, the length of the working fiber in single-use FURS can be kept shorter to ensure synchronization with the field of vision. In cases with a difficult anatomical structure of the lower calyx or incarcerated stone, we plan to “blind beat” for several seconds to loosen the stone, combined with the use of a set of stone baskets. The critical “few seconds” during surgery could reduce the likelihood of intraoperative transfer of PCNL. This study also showed that there was a high failure rate of lithotripsy in the subgroup with an IPA < 45°. However, transfer to a mini-PCNL decreased significantly after matching with the stone basket. The advantages of single-use FURS in the treatment of the lower pole stone often reduced the use of stone baskets. Our study found that the use of an intraoperative stone basket in single-use FURS was significantly lower than that for reusable FUR. This result confirmed the good deflection of single-use FURS. Razvan and collagues suggested that stone basket can be used to move lower pole stones and effectively improve the efficiency of lithotripsy and prolong the life of reusable flexible ureteroscopes [29]. We reported damage to five lens when using reusable FURS to treat lower pole stones and increased repair costs, while the lower use of stone baskets and zero repair costs for single-use FURS may be reduced economic costs. It is needed that more research about stone economics to prove this. To sum up, the advantage of single-use FURS increased the surgical success rate of lower pole stones and improved the calculi clearance rate,therefore we should choose SU-FURS in the treatment of lower pole stones.

This study had several limitations as it was a single-center, retrospective study on a relatively small number of patients. Although surgeons were on the same team, SU-FURS appeared later than R-FURS, potentially introducing a bias in technique. Fortunately, Our center was Shanxi center of China Urolithiasis Union, and the source of patients was facing Shanxi Province and surrounding areas. Secondly, as the information of SU-FURS group and R-FURS group were collected through hospital record, potential biases, if any, was unlikely to differ between two groups, meaning it was unlikely to skew the results in either direction in this study. Although we tried to minimize selection bias, the retrospective single-center design could limited the generalizability of our findings.. We will enlarge the sample size of SU-FURS group and R-FURS or ally member of China Urolithiasis Union in further study to verify the experimental results. Further more importantly, prospective, multi-center, controlled trials are needed to verify the conclusions.

## Conclusion

In summary, single-use and reusable FURS are alternative methods for treating lower pole stones (2 cm or less). Single-use FURS not only has a high success rate for lithotripsy, short operation time, and high stone clearance rate, but also when the anatomy of the lower calyx is difficult a single-use FURS should be preferable.

## Data Availability

No datasets were generated or analysed during the current study.

## Abbreviations

FURS: Flexible ureteroscopy
PCNL: Percutaneous nephrolithotomy
IPA: Infundibuloureter angle
SFR: The stone-free rate
IW: Infundibular width
IL: Infundibular length
BMI: Body mass index
ASA: Anesthesiology grade
IVP: Intravenous pyelography
RIRS: Retrograde intrarenal surgery
